# Susceptibility of bovine to SARS-CoV-2 variants of concern: insights from ACE2, AXL, and NRP1 receptors

**DOI:** 10.1186/s12985-023-02222-9

**Published:** 2023-11-27

**Authors:** Ying Ma, Mengyue Lei, Hongli Chen, Pu Huang, Jing Sun, Qiangming Sun, Yunzhang Hu, Jiandong Shi

**Affiliations:** 1 Yunnan Provincial Key Laboratory of Vector-Borne Diseases Control and Research, Institute of Medical Biology, Chinese Academy of Medical Sciences and Peking Union Medical College, 935 Jiaoling Road, Kunming, 650118 Yunnan Province China; 2 National Kunming High-Level Biosafety Primate Research Center, Institute of Medical Biology, Chinese Academy of Medical Sciences and Peking Union Medical College, 935 Jiaoling Road, Kunming, 650118 Yunnan Province China; 3 Kunming Medical University, Kunming, Yunnan Province China

**Keywords:** ACE2, AXL, NRP1, Cross-species transmission, Receptor, SARS-CoV-2 variants

## Abstract

The possibilities of cross-species transmission of severe acute respiratory syndrome coronavirus 2 (SARS-CoV-2) between humans and important livestock species are not yet known. Herein, we used the structural and genetic alignment and surface potential analysis of the amino acid (aa) in angiotensin-converting enzyme 2 (ACE2), tyrosine kinase receptor UFO (AXL), and neuropilin 1 (NRP1) in different species with substantial public health importance. The residues interfacing with the N-terminal domain (NTD) or receptor-binding domain (RBD) of S were aligned to screen the critical aa sites that determined the susceptibility of the SARS-CoV-2 to the host. We found that AXL and NRP1 proteins might be used as the receptors of SARS-CoV-2 in bovines. However, ACE2 protein may not be considered to be involved in the cross-species transmission of SARS-CoV-2 VOCs in cattle because the key residues of the ACE2-S-binding interface were different from those in known susceptible species. This study indicated that emerging SARS-CoV-2 variants potentially expand species tropism to bovines through AXL and NRP1 proteins.

## Introduction

According to WHO data, as of October 7, 2023, the number of confirmed cases of coronavirus disease 2019 (COVID-19) has exceeded 700 million, including 6,961,014 deaths, implying that COVID-19 has triggered a global public health crisis [1]. Syndrome coronavirus 2 (SARS-CoV-2) infection leads to the formation of clusters of severe respiratory illnesses resembling those caused by severe acute respiratory syndrome coronavirus (SARS-CoV) [2]. The clinical manifestations of COVID-19 encompass a wide spectrum, spanning from asymptomatic cases to severe illness and, in some instances, fatal outcomes [3]. While a significant proportion of individuals infected with SARS-CoV-2 may remain asymptomatic throughout the course of the infection, others may experience mild to moderate symptoms such as fever, cough, and fatigue [4]. Emerging research indicates that the circulation of SARS-CoV-2 may exert an impact on the infection patterns of human orthopneumovirus (HOPV) and respiratory syncytial virus (RSV) among individuals of varying age groups [5, 6]. The fatality rate of COVID-19 is predominantly influenced by age [7]. Continued research and surveillance are essential to monitor the impact of the virus on different age groups and inform targeted interventions to protect vulnerable populations, including young children.

A consensus shows that severe acute respiratory SARS-CoV-2 is a zoonotic spillover from bats into human populations through an intermediate host, such as raccoon dogs [8–10]. A plethora of variants already exist, including alpha, beta, gamma, delta, and omicron [11–14]. The transient acquisition of cross-species infectivity during the evolution of SARS-CoV-2 variants includes animal-to-human (zoonotic) and human-to-animal (zooanthroponotic) transmission, and even the potential spread in different animals.

The receptor-binding domain (RBD) and N-terminal domain (NTD) of the spike (S) protein are the important genetic determinants of the SARS-CoV-2 variant, leading to an increased risk of the worldwide transmission of COVID-19. Angiotensin-converting enzyme 2 (ACE2) receptor recognition by SARS-CoV-2 is a critical determinant of the host range and interspecies transmission [15, 16]. One study showed that the RBD can bind to bACE2 from *Rhinolophus macrotis* (bACE2Rm) [17]. Thus, the phenomenon of breaking the cross-species barrier may be closely associated with mutations in the RBD of the S protein that interacts with ACE2. Some animal species may contribute to the ongoing SARS-CoV-2 pandemic, even though the virus is primarily transmitted among humans [18]. Human-to-animal transmission of COVID-19 can propagate reinfections and lead to vaccine failures [19]. The latest research has shown that many mutations in the omicron variant are concentrated in the S protein (especially in the RBD region). According to recent studies, the original strain of SARS-CoV-2 was unable to infect wild-type mice [20, 21]. However, the Omicron variant has been found to have five adaptive mutation sites that may enable it to infect mice. Hence, the mutations of SARS-CoV-2 enhance the risk of cross-species transmission, leading to outbreaks in humans. Therefore, it is essential to investigate the mechanism underlying the breaking of the cross-species barrier from the perspective of zoonosis, which can provide new measures for the control of COVID-19.

Host cell receptor is a key determinant of viral tropism and disease outcomes. Recent studies have demonstrated that besides ACE2, tyrosine kinase receptor UFO (AXL) and neuropilin 1 (NRP1) also act as receptor proteins to mediate the SARS-CoV-2 invasion of cells [22–24]. AXL can promote viral infection of the human respiratory system and thus may serve as a potential target to help researchers develop future clinical intervention strategies [22]. Also, NRP1 can assist novel coronavirus to invade cells [25]. These studies suggested the role of ACE2, AXL, and NRP1 in the cross-species transmission of emerging SARS-CoV-2 variants.

To date, bovines are still one of the main sources of meat worldwide. The prevalence of COVID-19 may have potential short-, medium-, and long-term implications for food supply chains [26]. Bovines are important livestock species, and the prevalence of COVID-19 has a serious impact on bovine husbandry and slaughtering [27, 28]. Hence, the putative susceptibility of bovines to SARS-CoV-2 should be explored by examining the infectivity between bovines and humans. However, the influencing factors for SARS-CoV-2 infection in bovines are yet to be fully defined. Currently, various coronaviruses causing infections in bovines have been reported, such as bovine coronavirus (BCoV) and Human coronavirus OC43 (HCoV-OC43). The BCoV is a respiratory pathogen that can infect cattle and cause a variety of animal diseases [29]. Importantly, cases of the zoonotic transmission of BCoVs have been reported [30]. Hence, we hypothesize that the characteristics of SARS-CoV-2 suggest that it has the potential to infect cattle across species as it evolves and different variants emerge.

Previous studies have indicated that SARS-CoV-2 has the potential to be transmitted from various host species such as hamsters, minks, rhesus monkeys, ferrets, and mice. In recent times, a series of scholarly inquiries have disseminated revelations concerning the contagion of the SARS-CoV-2 within the ranks of wild white-tailed deer (*Odocoileus virginianus*), accompanied by the virus's autonomous metamorphosis occurring within the very fabric of their being [31, 32]. In addition, several studies have revealed that a diverse range of animals, including cats [33], dogs [34], and minks [35], can indeed become infected with SARS-CoV-2. These findings not only underscore the zoonotic potential of the virus but also serve as a stark reminder of its capacity for inter-species transmission. Such revelations shed light on the inherent risks associated with the cross-species dissemination of SARS-CoV-2.

However, the precise relationship between bovine ACE2, AXL, NRP1, and the S protein in terms of predicting infection capability remains unclear. Understanding the interplay and functional significance of these factors is crucial for comprehending the susceptibility of bovine species to SARS-CoV-2 and other related coronaviruses. Further investigation is warranted to elucidate the specific mechanisms by which these receptors and proteins contribute to viral entry and infection in bovines. By unraveling these intricate molecular interactions, we can enhance our knowledge of cross-species transmission and potentially develop effective strategies to mitigate the spread of zoonotic diseases.

Through structural and genetic analysis, we investigated the interaction between the S protein and its potential cellular receptors. Our findings indicate that SARS-CoV-2 may breach the species barrier and transmit to cattle. The interaction between bovine ACE2, AXL, NRP1, and S largely determined the cross-species transmission, thus providing clues for the potential recipient hosts. This study also revealed the important residues in NTD and RBD of S interacting with ACE2, AXL, and NRP1. These findings highlighted the cross-species transmission potential of SARS-CoV-2 and the risk of influence on beef supply and import. Public health measures, including virus surveillance, should be implemented to facilitate the control of the ongoing pandemic.

## Materials and methods

### Sequence analysis of ACE2, AXL, and NRP1 proteins

The isoforms of ACE2, AXL and NRP1 proteins from selected mammals were searched in UniProt (https://www.uniprot.org/). These ACE2, AXL and NRP1 proteins with their corresponding species are presented in Tables 1, 2 and 3. The sequence alignment analysis of the interface binding was performed between host receptors (ACE2, AXL and NRP1 proteins) and S of SARS-CoV-2 from minks, ferrets, rhesus monkeys, humans, mice, hamsters, and bovines. The clusterW and ESPript (https://espript.ibcp.fr/ESPript/cgi-bin/ESPript.cg) were used to compare the protein sequences of species.

**Table 1:** GenBank accession no. of ACE2 proteins used in this study

Host	GenBank accession no
Minks	XP_044091953.1
Ferrets	NP_001297119.1
Rhesus monkeys	NP_001129168.1
Humans	NP_001358344.1
Mice	NP_001123985.1
Hamsters	XP_003503283.1
Bovines	NP_001019673.2

**Table 2:** GenBank accession no. of AXL proteins used in this study

Host	GenBank accession no
Minks	XP_044113292.1
Ferrets	XP_004776133.1
Rhesus monkeys	XP_028695606.1
Humans	NP_068713.2
Mice	XP_006540052.1
Hamsters	XP_035292416.1
Bovines	XP_024834863.1

**Table 3:** GenBank accession no. of NRP1 proteins used in this study

Host	GenBank accession no
Minks	XP_044082878.1
Ferrets	XP_004774343.2
Rhesus monkeys	NP_001252745.1
Humans	XP_006717584.1
Mice	XP_0065430829.1
Hamsters	XP_007647231.1
Bovines	NP_001192589.1

### Structure simulation of receptor-S complex

The SWISS-MODEL (https://swissmodel.expasy.org/) was used to predict the tertiary structure of three different genes from humans, rhesus monkeys, hamsters, mice, ferrets, minks, and cattle. Electrostatic surface potential studies were performed using PyMol. The data on protein domains were obtained from the National Center for Biotechnology Information (NCBI) database (https://www.ncbi.nlm.nih.gov/Structure/cdd/wrpsb.cgi). The intracellular and extracellular distributions of proteins were explored and tested using TOPCONS (https://topcons.cbr.su.se/). Based on the structure of full-length human ACE2 with SARS-CoV-2 S RBD (PDB: 6M17), the structure of SARS-CoV-2 S RBD and ACE2 isoforms from selected mammals were simulated using SWISS-MODEL online server and analyzed using Chimera software version 1.14. The color intensities of the electrostatic surface potential were quantified using the ImageJ software.

## Results

### Structures of SARS-CoV-2 S protein complexed with ACE2 of bovines and six species

**Fig. 1:**
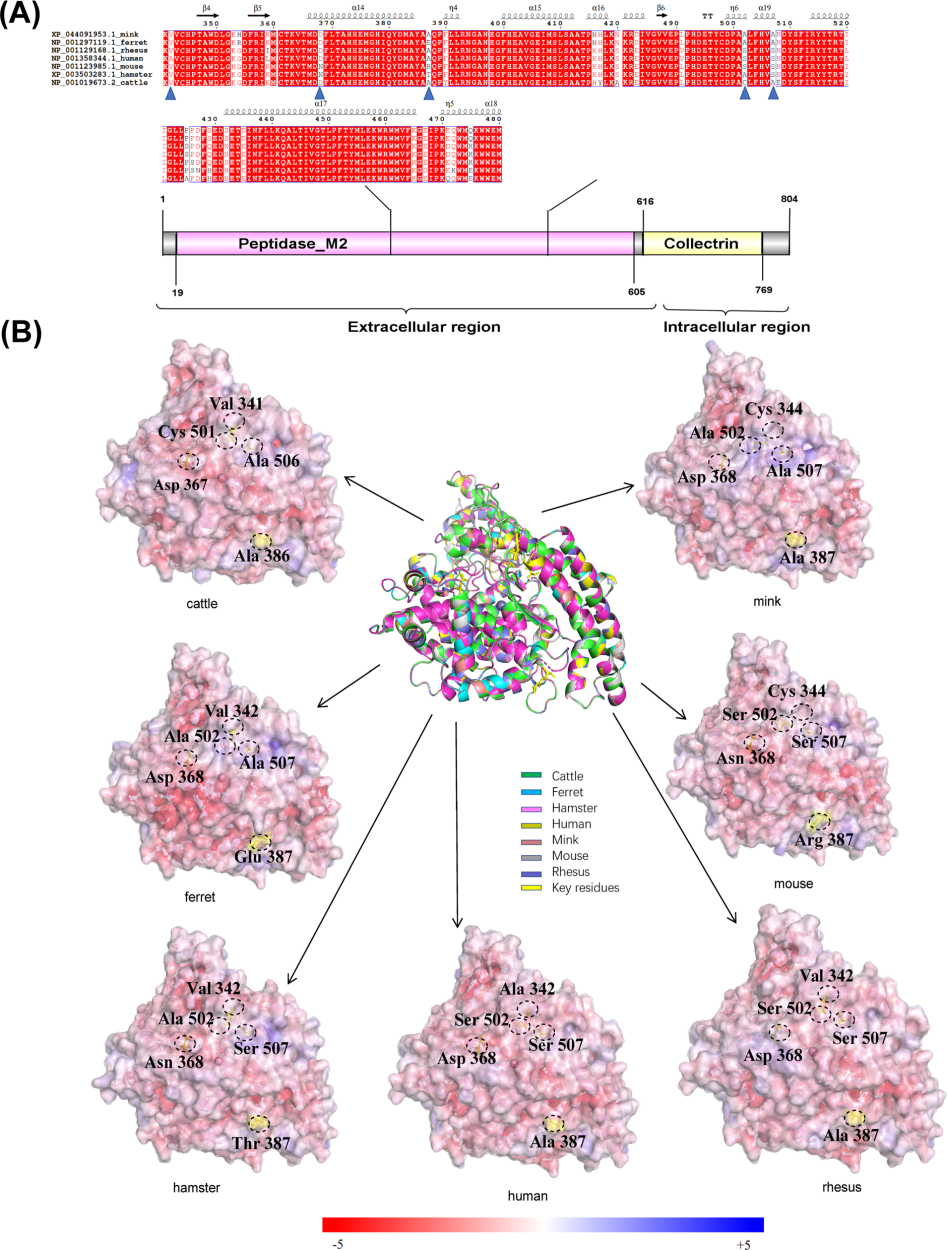
Alignment and electrostatic surface potential analysis of key amino acids residues of ACE2. **A** Sequence alignment analysis of the interface binding between ACE2 and S of SARS-CoV-2 from minks (GenBank accession no. XP_044091953.1), ferrets (GenBank accession no. NP_001297119.1), rhesus monkeys (GenBank accession no. NP_001129168.1), humans (GenBank accession no. NP_001358344.1), mice (GenBank accession no. NP_001123985.1), hamsters (GenBank accession no. XP_003503283.1) and bovines (GenBank accession no. NP_001019673.2). The ACE2 residues at positions 342, 368, 386, 502, and 507 are marked with *blue triangles*. **B** Electrostatic surface potential diagram of ACE2s in different species. The structural superposition of the ACE2 region 341–480 from cattle (*green*), minks (*brown*), ferrets (*cyan*), mice (*gray*), hamsters (*pink*), humans (*khaki*), and rhesus monkeys (*blue*) is in the center. The homology models of human ACE2 (PDBID 6m18) were used to compare the ACE2 structures of cattle, minks, ferrets, mice, hamsters, and rhesus monkeys. The tertiary structure was predicted using SWISS-MODEL. The *yellow sticks* highlight the five key differential residues of ACE2 binding with S of SARS-CoV-2. The details are *circled* using a *dashed line* in each electrostatic surface potential map. The electrostatic potential color range is –/ + 5

The sequence alignment was designed to show the changes in key residue sites between bovines and other species. According to the structure of the human ACE2-S RBD complex, Q24, D30, K31, E35, E37, D38, Y41, Q42, Y83, K353, and R393 were located in the interface and interacted with S RBD [36]. The key amino acid residues involved in the complex were located in the 342–507 segment of the ACE2 (Fig. 1A). As shown in Fig. 1A, the key residues of bovine ACE2 had weaker affinity characteristics compared with human ACE2. Among these sites, the mutation of A507S might influence the susceptibility of SARS-CoV-2 to cattle, while the V342A mutation was the same as that in rhesus monkeys, hamsters, mice, ferrets, and minks, indicating that this site mutation would not reduce the interface binding. Nevertheless, the mutation of the C502S locus appeared for the first time in five species and has predicted that porcine AXL protein is a potential receptor for SARS-CoV-2 [37]. We hypothesized that it might influence the potential of cross-species transmission in bovines by affecting the formation of hydrogen bonds. Specifically, the analysis of electrostatic surface potential showed differences in the key residues at the interface between ACE2 and S between bovine and other species (Fig. 1B). Based on these site changes, we hypothesized that ACE2 would not be the key receptor of SARS-CoV-2 cross-species infection. As a result, the local structural difference of ACE2s among different species suggested that bovine AXL might exhibit binding affinity toward S NTD similar to that of mink and ferret AXL.

The analysis of the electrostatic surface potential yielded intriguing insights into the kinetics of the RBD-ACE2 complex formation (Fig. 1B). As depicted in Fig. 1B, the surface potential of human ACE2 exhibited a negative charge. Based on this observation, we postulated that mutations in the RBD could potentially increase the positive surface charge, leading to a more positive surface potential of the RBD. Consequently, this could enhance the electrostatic attraction between human ACE2 and the RBD.

However, upon comparing the electrostatic surface potential of human ACE2 with that of bovine ACE2, we discovered that the Ala506 locus in bovine ACE2 resided within the positive region (blue region). This finding implies that the binding affinity of bovine ACE2 for S-RBD might be reduced, potentially impacting its interaction with SARS-CoV-2 variants. These observations shed light on the potential role of the Ala506 locus in modulating the virus-host interaction in bovine species.

### Structures of SARS-CoV-2 S protein complexed with AXL of bovines and six species

**Fig. 2:**
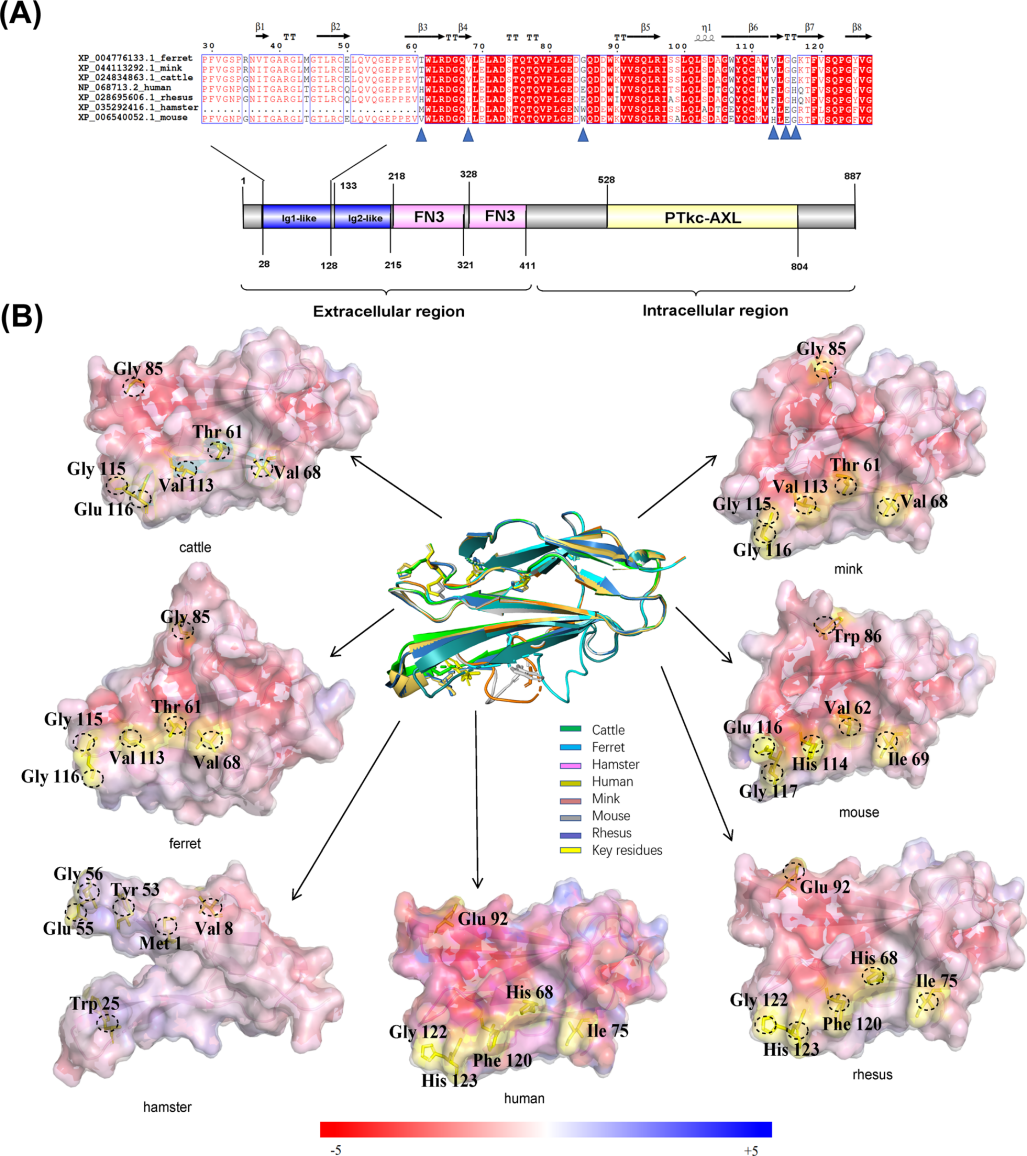
Alignment and electrostatic surface potential analysis of key amino acids residues of AXL. **A** Sequence alignment analysis of the interface binding between ACE2 and S of SARS-CoV-2 from ferrets (GenBank accession no. XP_004776133.1), minks (GenBank accession no. XP_044113292.1), bovines (GenBank accession no. XP_024834863.1), humans (GenBank accession no. NP_068713.2), rhesus monkeys (GenBank accession no. XP_028695606.1), hamsters (GenBank accession no. XP_035292416.1), and mice (GenBank accession no. XP_006540052.1). The ALX residues at positions 61, 68, 85, 113, 115, and 116 are marked with *blue triangles*. **B** Electrostatic surface potential diagram of AXLs in different species. The structural superposition of the AXL region 29–127 from cattle (*green*), minks (*brown*), ferrets (*cyan*), mice (*gray*), hamsters (*pink*), humans (*khaki*), and rhesus monkeys (*blue*) is in the center. The homology models of human AXL (PDBID 4yfg) were used to compare the AXL structures of cattle, minks, ferrets, mice, hamsters, and rhesus monkeys. The tertiary structure was predicted using SWISS-MODEL. The *yellow sticks* highlight the six key differential resides of AXL binding with S of SARS-CoV-2. The details are *circled* using a *dashed line* in each electrostatic surface potential map. The electrostatic potential color range is –/ + 5

As Fig. 2A showed, the results of sequence comparison showed that the key residues of bovine AXL had only E116G substitution compared with ferret and mink AXL, but were different from AXL in other species. The sequence in bovines also showed more substitutions and deletions at other residues compared with those in rhesus monkeys, mice, hamsters, and humans, but it was highly consistent with those ferrets and minks, indicating that bovines might be infected by SARS-CoV-2 through AXL protein, and the mode of infection was similar to that in ferrets and minks. This conjecture was also supported by the results of electrostatic surface potential analysis; the cattle, ferrets, and minks had highly similar patterns on the AXL-S interface. Figure 2B shows that the only difference between these species was that the Gly116 site was changed to Glu116.

Based on our analysis, we propose a hypothesis that SARS-CoV-2 may potentially undergo interspecies transmission via the AXL receptor, leading to the manifestation of COVID-19-like symptoms in cattle. This hypothesis stems from the inferred potential role of the AXL receptor in viral entry and infection. However, direct experimental evidence to support this hypothesis is currently lacking, necessitating further research. By delving into the mechanisms underlying the interaction between SARS-CoV-2 and the AXL receptor, we can gain a better understanding of the virus's interspecies transmission capabilities. This knowledge can contribute to the development of targeted strategies for the prevention and control of zoonotic diseases with cross-species transmission.

### Structures of SARS-CoV-2 S protein complexed with NRP1 of bovines and six species

**Fig. 3:**
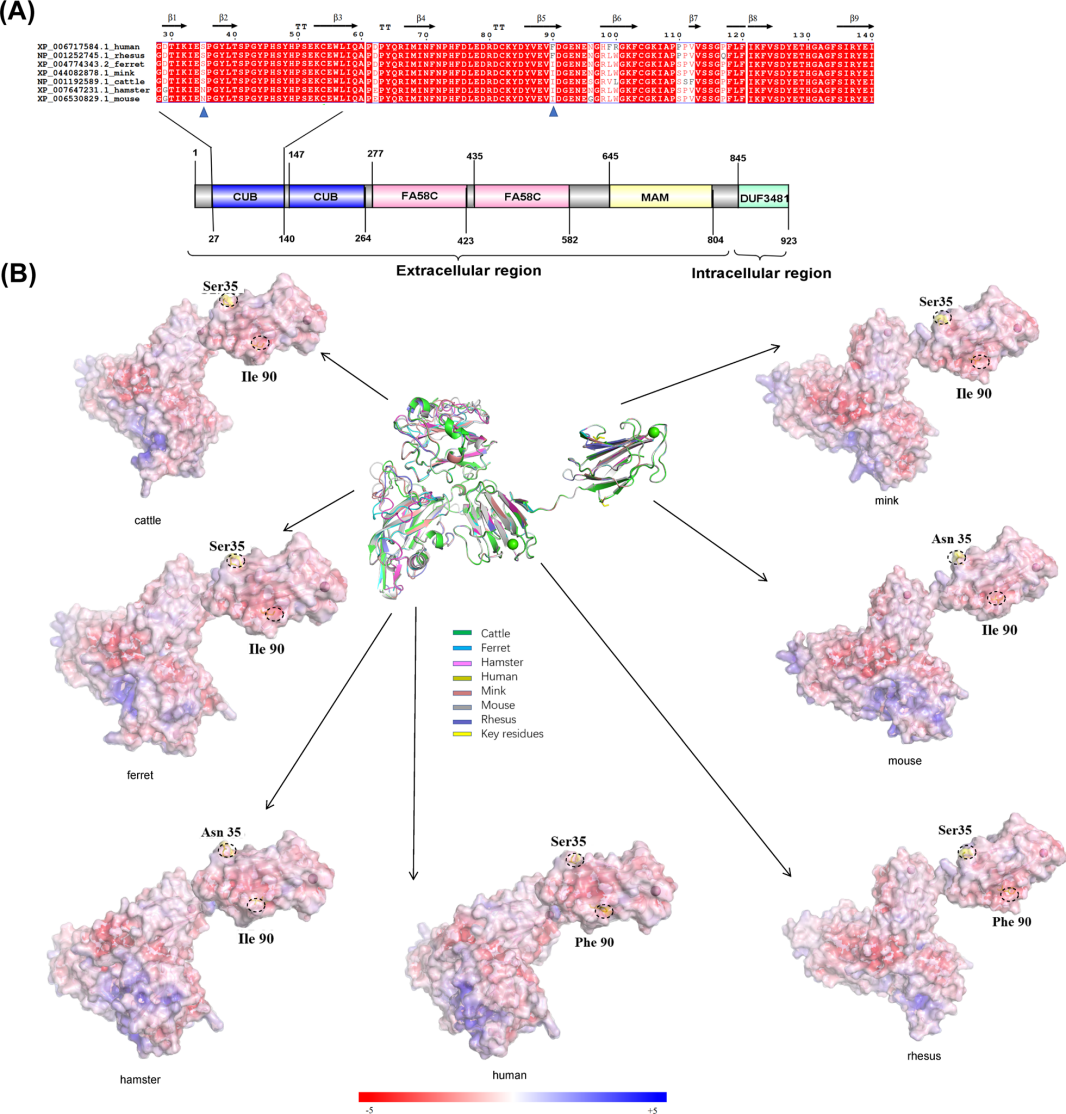
Alignment and electrostatic surface potential analysis of key amino acids residues of NRP1. **A** Sequence alignment analysis of the interface binding between NRP1 and S of SARS-CoV-2 from humans (GenBank accession no. XP_006717584.1), rhesus monkeys (GenBank accession no. NP_001252745.1), ferrets (GenBank accession no. XP_004774343.2), minks (GenBank accession no. XP_044082878.1), bovines (GenBank accession no. NP_001192589.1), hamsters (GenBank accession no. XP_007647231.1), and mice (GenBank accession no. XP_0065430829.1). The NRP1 residues at positions 35 and 90 are marked with *blue triangles*. **B** Electrostatic surface potential diagram of ACE2s in different species. The structural superposition of the NRP1 region 28–140 from cattle (*green*), minks (*brown*), ferrets (*cyan*), mice (*gray*), hamsters (*pink*), humans (*khaki*), and rhesus monkeys (*blue*) is in the center. The homology models of human NRP1 (PDBID 7m0r) were used to compare the NRP1 structures of cattle, minks, ferrets, mice, hamsters, and rhesus monkeys. The tertiary structure was predicted using SWISS-MODEL. The *yellow sticks* highlight the two key differential resides of NRP1 binding with S of SARS-CoV-2. The details are *circled* using a *dashed line* in each electrostatic surface potential map. The electrostatic potential color range is –/ + 5

We also predicted the NRP1 protein as a receptor in SARS-CoV-2 to analyze the possibility of the bovines being infected by SARS-CoV-2 across other species. Similar to the results in Fig. 2A, we found that cattle, ferrets, and minks were still highly similar in sequence, with no change in the position of key residues (Fig. 3A). Based on these results, we preliminarily considered that both NRP1 and AXL were potential receptors of SARS-CoV-2 in bovine infection. As anticipated, the findings from the analysis of electrostatic surface potential provided support for the hypothesis that ferrets and minks share similarities with bovines in terms of the binding interface between the spike protein (S) and the receptor-binding domain (RBD). This observation suggests that these animal species may exhibit comparable binding characteristics and interactions with SARS-CoV-2 (Fig. 3B).

### Key residues in the receptor-S RBD/NTD complexes

**Fig. 4:**
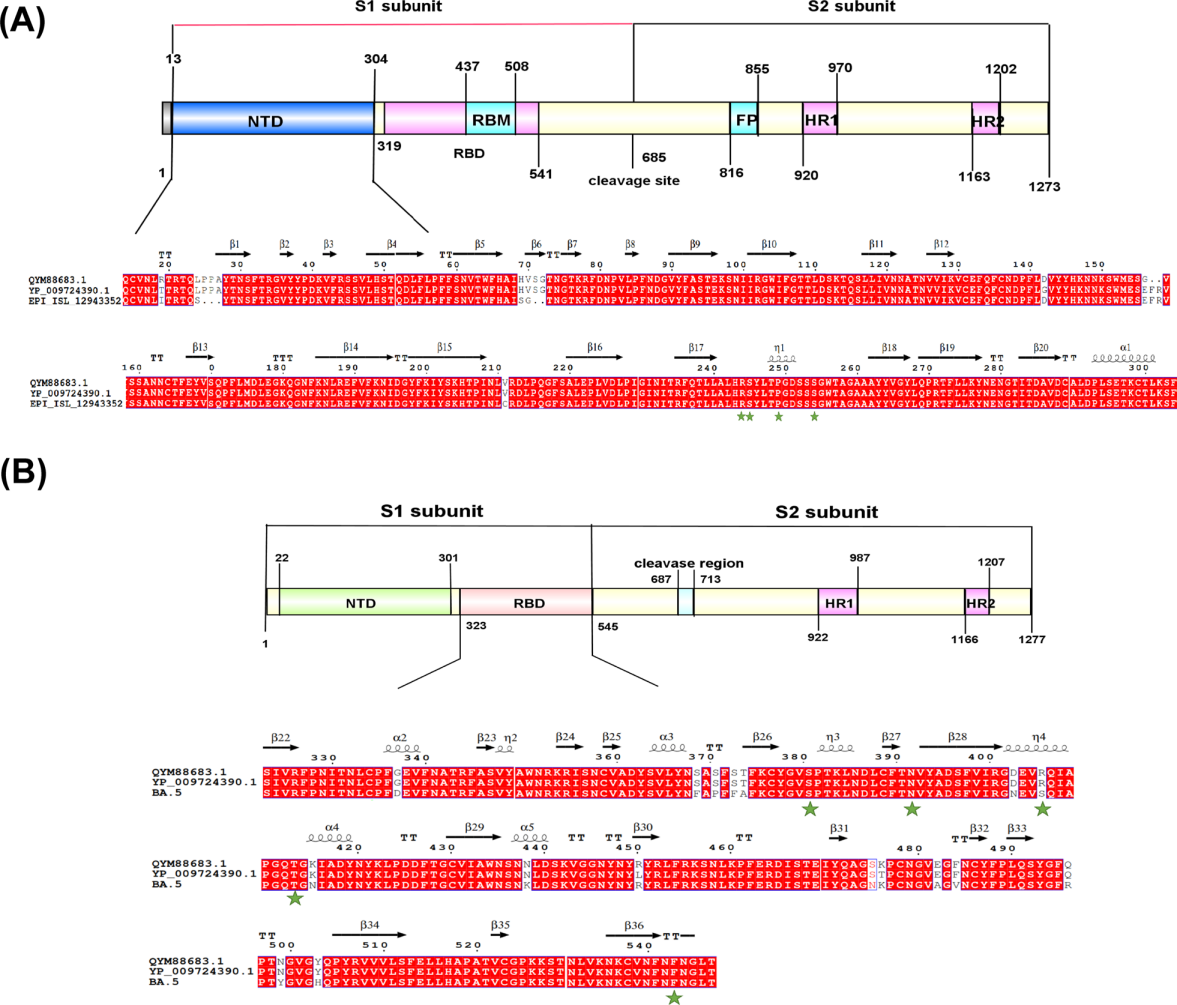
Sequence alignment of S protein from three SARS-CoV-2 strains, including Wuhan-Hu-1 (GenBank accession no. YP_009724390), delta variant (GenBank accession no. QYM88683), and omicron variant (GenBank accession no. UFS23237). **A** The important residues in RBD of S interacting with ACE2 and NRP1 are marked with *green stars*. **B** The important residues in NTD of S interacting with AXL are marked with *green stars*

The SARS-CoV-2 genome is approximately 30,000 nucleotides long and contains the largest single-stranded positive-sense RNA. SARS-CoV-2 variants of concern such as alpha, beta, gamma, delta, and omicron were identified by the WHO in February 2022 [38]. Among these, omicron and delta variants had the stronger receptor-binding ability [37]. The aa sequences were compared and analyzed r to clarify the influence of some changes in S NTD of different SARS-CoV-2 variants. The results revealed that the key residues R244, S245, P249, and S254 in the ACE2 and NRP1-S NTD and S381, N392, T413, and F543 in the AXL-S RBD complexes were unaltered (Fig. 4). We conjectured that the R406S mutation in the omicron variant may affect the ability of SARS-CoV-2 to cause COVID-19 in various species, including humans, mice, and cattle. If these variants become more infectious in cattle, the potential for zoonotic transmission may become more complicated. The delta and omicron variants have more mutation sites and are therefore more infections than other variants. They also have different pathological features in the clinic. We compared and analyzed their aa sequences to explain the effect of these two variants in S NTD or RBD of SARS-CoV-2. The results showed that the important residues of the omicron variant in the ACE2-S of RBD were replaced at R406 (Fig. 4A).

As depicted in Fig. 4B, the critical residues within the NTD that interacted with the AXL receptor were identical in both the delta and omicron variants, with fewer mutations observed in other sites. Notably, only one mutation (R406) was detected in the crucial residue of the RBD involved in the interaction with NRP1 (Fig. 4A). However, the presence of amino acid variations does not definitively indicate that NRP1 is not utilized as a receptor by SARS-CoV-2 in infected cattle. To gain further insights, electrostatic potential energy analysis was conducted, revealing similarities between bovines and other susceptible species. Our hypothesis suggests that amino acid mutations have the potential to influence viral evolution and outcomes, thereby impacting the infectivity of SARS-CoV-2, including an increased likelihood of cross-species transmission of SARS-CoV-2 variants.

## Discussion

The current public health security of human society is threatened by various highly contagious viruses, including well-known ones such as Influenza, Human Immunodeficiency Virus-1 (HIV-1), Ebola, SARS-CoV-2, as well as the emerging monkeypox outbreak that has surfaced alongside the subsiding COVID-19 crisis. Ebola virus disease (EVD) is a highly contagious and often fatal illness caused by the Ebola virus, which induces severe hemorrhagic fever in humans and other primates [39]. The HIV-1 primarily targets the human immune system, leading to subsequent infections and diseases, making it one of the most devastating pandemics in history [40]. Monkeypox, caused by a virus belonging to the *poxviridae* family, currently lacks definitive treatment options. It is a virus that can be transmitted between animals and humans, giving rise to zoonotic diseases [41].

Zoonotic diseases have been responsible for recurring outbreaks among human populations on a global scale [42]. A study from 2021 highlighted that Bangladesh has documented several overlooked yet potentially zoonotic diseases, including highly pathogenic avian influenza (HPAI), nipah virus (NiV), Anthrax, and COVID-19 [43]. Although extensive studies have been conducted on the natural and intermediate hosts of SARS-CoV-2, the possibilities of cross-species transmission of SARS-CoV-2 between humans and important livestock species are not yet known. Recent studies have demonstrated that pigs and chickens are not susceptible to SARS-CoV-2 [44]. However, whether it can be transmitted between cattle, which are also a major source of meat supply, and other species is still unclear. A study has demonstrated that SARS-CoV-2 is capable of infecting the respiratory tissues of cattle and sheep in ex vivo organ cultures, while pig tissues do not allow for the replication of the virus [45]. Although in vivo experimental infections research results indicated that bovines are not susceptible to SARS-CoV-2 [46]. But our study also proposes the age, management, and general health of the cows must be considered when assessing the risk of viral infection in a herd. Notably, a recent study reported that cows from different herds in Germany were seropositive for SARS-CoV-2 [47], suggesting that although overall cattle susceptibility appears to be low, spillover events are still possible for this species might happen. Based on the available data, it is crucial to conduct a thorough examination of the impact of natural SARS-CoV-2 infection on ruminant farms. Additionally, it is important to investigate the potential presence of the virus in slaughterhouses, as there is a risk of transmission to personnel that must be considered [48]. Specifically, it is ascertained that the interaction between receptors of SARS-CoV-2 in bovines and humans can prevent the impact of cross-species transmission on health and economy in earlier stages of the next global pandemic or serve as research models for COVID-19 [49]. So we aimed to identify ACE2, AXL, and NRP1 as an entry receptor to explore the possibility of SARS-CoV-2 infecting bovine. It is possible to provide useful evidence about sequence and structure studies in future SARS-CoV-2 cross-species infections.

Based on the aforementioned information, we conducted a screening of the key residues within the ACE2, AXL, and NRP1 receptors that interact with the RBD or the NTD of the S protein in bovines and various other species. This screening involved a comparison of amino acid sequences. By analyzing these sequences, we aimed to identify and characterize the specific residues that play a crucial role in the binding interface between the receptors and the S protein.

This comparative analysis provides valuable insights into the conservation or variation of key residues across different species, shedding light on potential differences in the receptor-virus interactions and susceptibility to SARS-CoV-2 infection. Understanding the variations in these binding interfaces is essential for elucidating the mechanisms underlying viral entry and infection, as well as for predicting the potential for cross-species transmission. Further investigations are needed to decipher the functional implications of these identified residues and their contributions to the overall infectivity and pathogenesis of SARS-CoV-2 in different hosts. Such knowledge can aid in the development of targeted interventions and preventive measures to mitigate the spread of the virus.

As illustrated in Figs. 1B, 2B, and 3B, the electrostatic potential energy map predicted the susceptibility of bovines to cross-species infection by SARS-CoV-2 and also whether ACE2, AXL, and NRP1 proteins might serve as potential receptors for SARS-CoV-2. The strength of intracellular protein electrostatic interactions was related to the free energy of protein folding transfer, and electrostatic interactions were important for protein binding. Therefore, the analysis of the electrostatic surface potential could predict the ability of proteins to interact with each other. We focused on three proven SARS-CoV-2 receptors that could help the virus invade cells to trigger an infection in the organism, including various emerging variants. SWISS-MODEL was used to predict protein tertiary structures to determine the key aa of binding sites between different species and to analyze the degree of similarity of key receptor-S protein binding interfaces associated with SARS-CoV-2-infected cells between humans, rhesus monkeys, hamsters, mice, ferrets, minks, and cattle.

To date, a large number of studies have focused on targeting the ACE2 receptor. It has been suggested that some animals may have mutations in the ACE2 receptor–binding domain, which prevents SARS-CoV-2 from binding to ACE2 [50, 51], making them less susceptible to the virus. There are many reports on the interaction between bovine ACE2 and SARS-CoV-2. A study on functional assessment of ACE2 orthologs mediating SARS-CoV-2 virus entry showed that ACE2 of cattle orthologs can indeed mediate SARS-CoV-2 entry [52]. Meanwhile, a study showed that the infectivities of SARS-CoV-2 variants were not alerted in ACE2-overexpressed cell lines from cattle [53]. This study showed that the bovine ACE2 and S RBD binding interface was altered at the location of key residues. However, ACE2 proteins from members of Bovidae and Cricetidae retained most of the key residues in ACE2 associated with the RBD of SARS-CoV-2, allowing them to associate with the S RBD of SARS-CoV-2 [16]. We suggested that species distinctions in Bovidae may have implications, as *Bos taurus* cannot represent all bovids in Bovidae, which also includes *B. inducus*, *B. taurus*, and *B. mutus*. At the same time, a study comparing the interaction surfaces of human ACE2 and bovine ACE2 showed that there were two types of aa changes in cattle [54]. Based on previous research reports, our analysis of key residues of ACE2, and the predicted structure of electrostatic surface potential, we believe that the ACE2 receptor may not mediate the cross-species transmission of SARS-CoV-2 between cattle and other species. The AXL protein is a potential receptor for the neocoronavirus infection of the human respiratory system and can participate in SARS-CoV-2 infection independent of the ACE2 receptor [22, 55].

In studies on AXL proteins, we confirmed that bovines had a pattern highly similar to that in ferrets and minks at the AXL-S interface (only changing the locus Gly116 to Glu116), but the pattern differed significantly from that in mice, hamsters, rhesus monkeys, and humans. The comparative results of the tertiary structures indicated that bovine AXL had binding similar to that of ferret and mink AXL for the S NTD. Some studies suggested that few patients with COVID-19 had neuropsychiatric symptoms and other patients had varying degrees of encephalitis, which was because astrocytes in the central nervous system were attacked by NRP1 receptors after SARS-CoV-2 infection [56, 57]. Consequently, using the same analysis method, we found that the electrostatic potential energy of the NRP1-S binding interface in bovines was the same as that in ferrets and minks (Ser35 and He90), indicating that bovines might be as susceptible to SARS-CoV-2 infection as ferrets and minks. More detailed pathological analysis and mechanistic studies can be carried out in the future using AXL and NRP1 proteins as receptors.

In this study, we highlighted the sequence of the original strain of Wuhan infection; the most infectious delta variant strain and the most variable omicron variant strain were compared and analyzed. The results in Fig. 4 showed the key residues of both ACE2-S RBD complex and NRP1-S RBD complex of the two variants were identical, while the mutations AXL-S NTD complex of key residues in R406 of omicron variant strains. Moreover, these two variants showed deletion or substitution of residues in multiple identical positions. The difference in aa residues might lead to changes in the infectious ability and infectivity of SARS-CoV-2, thus affecting the disease phenotypic characteristics of COVID-19 in cattle. If mutations in the key residues of SARS-CoV-2 variants enhance the potential for the cross-species transmission of the virus, the time when these mutants infect cattle, the increase in the risk of zoonosis, and the direction of virus evolution will be more difficult to predict.

Our predictive analysis of the receptors of SARS-CoV-2 infected bovines showed that AXL and NRP1 proteins most likely promoted the cross-species transmission of SARS-CoV-2 between bovines and other species. On the contrary, the ACE2 protein may not mainly mediate the invasion of SARS-CoV-2. The predictive analysis of ACE2, AXL, and NRP1 receptor binding of SARS-CoV-2 in bovines and other susceptible species indicated the risk of the cross-species transmission of the virus in domestic animals. The findings of this study might be of great significance in controlling SARS-CoV-2 infection and preventing pandemics among livestock caused by COVID-19.

Undoubtedly, there is an urgent need for further exploration of the specific molecular mechanisms underlying the interactions of ACE2, AXL, and NRP1 receptors with SARS-CoV-2. While we have already investigated the binding interfaces between these receptors and the virus through comparative analysis of amino acid sequences, a more comprehensive understanding of the detailed mechanisms governing these interactions is still required. Additional investigations can be conducted using functional experiments and other methodologies to elucidate the binding modes between the receptors and the virus, the interplay of amino acid residues, and the key factors contributing to infectivity. Furthermore, the use of in vitro and in vivo models can be considered to validate the biological effects of these interactions and evaluate their variations across different species.

Through in-depth exploration of these molecular mechanisms, we can enhance our understanding of the viral entry pathways, host specificity, and potential for cross-species transmission of SARS-CoV-2. This knowledge is of paramount importance in formulating effective strategies for disease prevention and control, ultimately aiding in the mitigation of viral spread and the safeguarding of public health.

## Conclusions

Zoonotic diseases, also referred to as diseases naturally transmitted between vertebrate animals and humans, pose a significant public health threat. These diseases have gained attention due to their high infectivity and their role in causing infectious disease outbreaks in human populations. In recent times, the emergence of SARS-CoV-2, the virus responsible for the COVID-19 pandemic, has further highlighted the global threat posed by zoonotic diseases. In this study, we focused on bovines as a potential host for the virus and investigated the receptor proteins involved in viral attachment and entry. Through our analysis, we identified AXL and NRP1 as potential receptors for SARS-CoV-2 in bovines. Interestingly, we also observed that the ACE2 protein, which serves as the primary receptor for SARS-CoV-2 in humans, may not be involved in the cross-species transmission of SARS-CoV-2 in cattle. This discrepancy is likely due to differences in the key residues within the ACE2-S binding interface between cattle and known susceptible species. These findings underscore the significance of studying the effects of amino acid changes on the binding interactions between the virus and its receptors. Investigating the specific mechanisms by which these mutations influence the affinity and stability of the virus-receptor complexes is crucial for comprehending the dynamics of SARS-CoV-2 transmission across different species. Furthermore, elucidating the potential implications of these variations in terms of viral infectivity and the emergence of novel variants can inform the development of targeted strategies for disease control and prevention. Additional research is warranted to unravel the intricate interplay between viral mutations, receptor utilization, and the potential for interspecies transmission of SARS-CoV-2 VOCs.

## Data Availability

The datasets used and/or analysed during the current study are available from the corresponding author on reasonable request.
